# Proapoptotic and antimigration properties of osthole in combination with LY294002 against human glioma cells

**DOI:** 10.1007/s00210-024-03424-w

**Published:** 2024-10-01

**Authors:** Joanna Sumorek-Wiadro, Justyna Kapral-Piotrowska, Adrian Zając, Aleksandra Maciejczyk, Monika Hułas-Stasiak, Krystyna Skalicka-Woźniak, Wojciech Rzeski, Bożena Pawlikowska-Pawlęga, Joanna Jakubowicz-Gil

**Affiliations:** 1 Department of Functional Anatomy and Cytobiology, Institute of Biological Sciences, Maria Curie-Sklodowska University, Akademicka 19, 20-033 Lublin, Poland; 2 Independent Laboratory of Natural Products, Medical University of Lublin, Chodzki 1, 20-093 Lublin, Poland; 3 Department of Medical Biology, Institute of Rural Health, Institute of Agricultural Medicine, Jaczewskiego 2, 20-950 Lublin, Poland

**Keywords:** Glioma, Osthole, LY294002, Migration, Cell death

## Abstract

Anaplastic astrocytoma and glioblastoma multiforme are infiltrating and vascularized gliomas with a high degree of chemoresistance and metastasis. Our previous studies have shown that osthole may be of great importance in the treatment of gliomas. Therefore, in this work, for the first time, coumarin was used in combination with LY294002—an inhibitor of the PI3K-Akt/PKB-mTOR pathway, which is overly active in gliomas. MOGGCCM and T98G cells were incubated with osthole and LY294002, alone and in combination. Staining with specific fluorochromes was used to visualize cell death and the scratch test to assess the migration. The level of proteins was estimated by immunoblotting. Forming protrusions were visualized by SEM, and immunocytochemistry was used to determine the localization of proteins. Additionally, the expression of Bcl-2, beclin 1 and Raf kinase was silenced using specific siRNA. The obtained results showed that osthole in combination with LY294092 effectively inhibited the migration of glioma cells by reducing the level of metaloproteinases and Rho family proteins, as well as decreasing the level of N-cadherin. In addition, the combination of compounds induced apoptosis. New combination of compounds shows a high pro-apoptotic potential and also inhibits the migration of gliomas cells.

## Introduction

Osthole (7-methoxy,8-isopentenyl coumarin) is a naturally occurring representative of the simple coumarins, substituted with methoxy and isoprenyl moieties at the C7 and C8 positions, respectively. Due to the wide spectrum of biological activity, this compound is of great research interest. So far, its beneficial effect on the central nervous system (anticonvulsant effect), circulatory system (anticoagulant effect) and skeletal system (treatment of osteoporosis) has been demonstrated. Its anti-inflammatory, anti-allergic and antiviral effects have also been proven. In addition, it has a cytotoxic and antiproliferative effect on cancer cells, and also reduces angiogenesis and prevents the formation of metastases to other tissues. Due to its high anticancer activity, osthole may therefore be a potential drug against many types of cancer. According to in vitro studies, coumarin has anticancer activity against lung cancer (A549), stomach cancer (HGC27 and SGC-7901), leukemia (P-388 D1), breast cancer (MCF-7, MDA-MB 231, MDA-BT-549, MDA-MB-468 and MDA-MB 435), medulloblastoma (TE671), laryngeal cancer (RK33) and cervical cancer (HeLa) (Dai et al. 2018; Xu et al. 2018, 2011; Wang et al. 2015; Jarząb et al. 2014; Yang et al. 2010; Chou et al. 2007). Our recent studies have shown that ostol effectively induces apoptosis in glioma cells (MOGGCCM and T98G cell lines). What is more, it enhances the anticancer effect of chemotherapeutic agents, such as temozolomide or sorafenib (Sumorek-Wiadro et al. 2020a, 2020b).

Gliomas are tumors of the central nervous system that account for about 70% of all brain tumors. Among them, anaplastic astrocytoma (AA) and glioblastoma multiforme (GBM) have the most aggressive course. This infiltrating and vascularized tumors with a high degree of metastasis are practically impossible to remove completely surgically and the implemented treatment allows only to improve the patient’s comfort and life extension, which in the case of AA and GBM does not exceed 5 and 2 years from the moment of diagnosis, respectively. In addition to the difficult location, a big problem is their ability to acquire resistance to radio- and chemotherapy. High mortality is also associated with their potential for rapid growth and the formation of distant metastases (Armento et al. 2017; Langhans et al. 2017). As it is known from the literature, in the vast majority of gliomas, changes in signal transmission from the cell membrane to the nucleus through intracellular signaling pathways, including PI3K-AKT/PKB-mTOR, are observed (Ludwig and Kornblum 2017). Its excessive activation leads to increased proliferation of cancer cells, promotes their survival and stimulates angiogenesis. It has been observed that this pathway also plays an important role in the acquisition of resistance to applied therapies and its activation is observed in the majority of secondary gliomas not susceptible to TMZ treatment (Haas et al. 2018; Steelman et al. 2011). Therefore, the development of new therapeutic strategies that take into account the blocking of the activity of this pathway by specific inhibitors may be of great importance in the treatment of these tumors. It has been described so far that the inhibition of transmission by PI3K-AKT/PKB-mTOR increases the sensitivity of glioblastoma cells to the induction of programmed death, and combination therapy, especially with natural compounds, enhances the anticancer potential of clinically used treatment (Jakubowicz-Gil 2009; Schwartzbaum et al. 2006). Our recent studies have shown that silencing of PI3K protein expression by specific siRNAs significantly increases the pro-apoptotic activity of oshtole in MOGGCCM and T98G cells (Sumorek-Wiadro et al. 2020b). Our recent studies have shown that the morpholino derivative of quercetin—LY294002—is an effective inhibitor of PI3K kinase. This compound induces apoptosis and autophagy in human glioblastoma multiforme (T98G) and anaplastic astrocytoma (MOGGCCM) cells (Zając et al. 2021). LY294002 has also been shown to reduce tumor invasiveness by inhibiting the expression of MMP 2 and increasing the level of early growth response factor 1 (EGR-1) (Liu et al. 2008). Therefore, in this study, we attempted to evaluate the simultaneous application of coumarin with the PI3K kinase inhibitor—LY294002—on the induction of programmed death and migration potential in human anaplastic astrocytoma and glioblastoma multiforme cells. In order to understand the mechanism of action of the tested compounds, their influence on the level of proteins involved in these processes was determined.

## Materials and methods

### Cells and culture conditions

Human glioblastoma multiforme (T98G) and human anaplastic astrocytoma (MOGGCCM) cell lines were purchased from the European Collection of Cell Cultures (ECACC, 86022702) and American Type Culture Collection (ATCC, CRL-1690), respectively. The culture medium consisted of a 1:1 (MOGCCM) or 3:1 (T98G) mixture of DMEM (Dulbecco’s minimal essential medium, Sigma) and the nutrient mixture Ham F-12 (Sigma) supplemented with 10% fetal bovine serum (FBS), 100 IU/mL penicillin (Sigma) and 100 μg/mL streptomycin (Sigma). All the cells were incubated in a 37 °C with 5% CO_2_ and 95% humidity.

The studies also used the oligodendrocyte cell line OLN-93 (obtained from the Department of Neonatology, Charite, Campus Virchow Klinikum, Humboldt University of Berlin). The culture medium was a mixture of DMEM:F-12 in ratio 1:1 with 10% FBS and 100 µg/mL penicillin and streptomycin.

### Osthole isolation

Isolation of the osthole from the plant material was carried out according to the method described by Jarząb et al. (Jarząb et al. 2014). Celery fruits (*Cnidum monnieri L.*) were dried at room temperature, then pulverized and extracted with petroleum ether (50 g in 500 mL) for 30 min. The procedure was repeated three times, then excess solvent was removed using a rotary evaporator. For further purification compound, the HSCCC high-performance counter-current chromatography method was used with the two-phase solvent systems made of *n*-heptane, ethyl acetate, methanol and water (HEMWat) in volume ratio 3:2:3:2. After the analytical column is completely filled with the stationary phase, the mobile phase was pumped in (flow rate 1 mL /min) simultaneously with the apparatus rotated at 1600 rpm. After reaching hydrodynamic equilibrium, through the injection valve with a capacity of 1 mL, the dissolved extract was introduced to the column in a two-phase solvent system (60 mg in 1 mL). The retention in the solid phase was 70%. The column effluent was monitored continuously with a UV detector at 320-nm separation of 600 mg of the extract yielded 2 mg of the target compound. Identification of the isolated compound was performed by comparing the retention time UV-DAD spectra with spectra obtained according to standards under the same conditions.The purity of the osthole, determined by HPLC-DAD (high-performance liquid chromatography with diode-array detection), was 99%.

### Drug treatment

MOGGCCM and T98G cells were incubated with osthole and LY294002 (Sigma-Aldrich) at final concentrations estimated on the basis of previous experiments (150 and 10 µM, respectively) for 24 h in single and simultaneous application (Zając et al. 2021; Sumorek-Wiadro et al. 2020a). Control MOGGCCM and T98G cells were incubated with 0.01% DMSO only.

### Microscopic detection of apoptosis, autophagy and necrosis

To identify apoptotic and necrotic cells, fluorochromes:propidium iodide (starting concentration 0.5 mg/mL) and Hoechst 33342 (starting concentration 0.4 mg/mL) were used (Allen et al. 2001). Cell cultured on eight-well glass slide Lab-TekTM (NuncTM, ThermoFisher), after incubation with appropriate compounds, were stained with a solution of propidium iodide and Hoechst 33342 in distilled water in a ratio of 3:2:5, and incubated in the dark at 37 °C for 5 min.

Orange acridine staining was used to visualize autophagic cells (Thomé et al. 2016). Studied cells cultured on eight-well slide LabTek were incubated in the dark with 5% acridine orange for 15 min at 37 °C and rinsed three times with PBS.

Stained cells on microscopic slides were analyzed under confocal microscope Axiovert 200M with LSM 5 PASCAL scanning head (Zeiss) at a wavelength of 420 nm (apoptosis and necrosis) and 526 m (autophagy).

### Western blotting analysis

Cellular protein expression was assessed by western blotting. Cells, after a 24-h incubation with osthole and/or LY294002, were transferred to Eppendorf tubes and centrifuged (12,000 × G, 10 min). The supernatant was then discarded, cell pellet resuspended in buffer (125 mM Tris-HCl pH 6.8; 4% SDS; 10% glycerol; 100 mM dithiothreitol) and boiled for 10 min. After centrifugation at 12,000 g for 10 min, the supernatants were collected. Protein concentration in the obtained cell-free extracts was determined by the Bradford method (Bradford 1976). In total, 80 μg of proteins was separated by 10% SDS-PAGE and electroblotted onto Immobilon-P PVDF membrane (Sigma). After blocking with 5% low fat milk for 1 h, membranes were incubated overnight at 4 °C in a solution of mouse primary antibodies: anti-caspase 3 (Santa Cruz Biotechnology, sc-56053, 1:1000), anti-beclin 1 (Santa Cruz Biotechnology, sc-48341, 1:500) and anti-Cdc 42 (Santa Cruz Biotechnology, sc-8401, 1:200), anti-Rac 1 (Santa Cruz Biotechnology, sc-217, 1:200), anti-N-cadherin (Santa Cruz Biotechnology, sc-59987, 1:200), anti E-cadherin (Santa Cruz Biotechnology, sc-59778, 1:200), anti-MMP-2 (Santa Cruz Biotechnology, sc-13595, 1:200) and anti-Rho A (Santa Cruz Biotechnology, sc-166399, 1:100), anti-MMP-9 (Cell Signaling Technology, 3852, 1:1000).

The membranes were then washed three times (10 min) in PBS enriched with 0.05% Triton X-100 (Sigma), followed by for 2-h incubation in a solution of secondary anti-mouse antibodies (dilution 1: 30,000), conjugated with alkaline phosphatase. After this time, the membranes were rinsed twice in PBS (10 min). For the visualization of the measured proteins, alkaline phosphatase substrates: 5-bromo-4-chloro-3-indolylphosphate (BCIP) and nitro-blue tetrazolium (NBT) (Sigma) in N,N-dimethylformamide (DMF, Sigma) were used. The obtained membranes were subjected to quantitative analysis using the ImageJ program (Scion Corporation, USA). The data were normalized relative to β-actin (Santa Cruz Biotechnology, sc-517582, 1:2000). Three independent experiments were performed.

### Cell transfection

The expression of genes encoding Bcl-2 protein, beclin 1 and Raf kinase was blocked using specific siRNAs. Cells at the density of 2 × 105 cultured on eight-well slides Lab-TekTM (NuncTM, ThermoFisher) were washed three times with DMEM:F-12 Ham medium (3:1), without serum and antibiotics. The blocking mixture, containing 2 μL of specific anti-Bcl-2, anti beclin 1 or anti-Raf siRNA (Santa Cruz Biotech Dallas, TX, USA), 2 μL of transfection reagent (Santa Cruz Biotech Dallas, TX, USA) and 250 μL of transfection medium (Santa Cruz Biotech), free of serum and antibiotics, was then added to each well. After 5-h-long incubation, the blocking mixture was supplemented with medium (250 mL) with 20% bovine serum and 200 μg/mL of antibiotics. After 18 h the medium was replaced with a new one (DMEM:F-12 Ham 1:1, 10% FBS, 100μg/mL antibiotics), and transfected cells were incubated for 24 h with test compounds and indirectly used for further analyses.

### Immunocytochemistry

Control, as well as osthole and LY294002 treated cells, was grown in the eight-well glass slide Lab-Tek™ chambers in 1 × 10^5^ seeding density. The cultures were washed three times with PBS with Ca^2+^ and Mg2^+^ and fixed 3.7% cold paraformaldehyde for 10 min, and treated with 0.2% Triton X-100 solution for 10 min in room temperature. Then, the cultures were incubated for 30 min with 5% bovine serum albumin, washed with PBS buffer and exposed for mouse monoclonal primary anti-Cdc 42, anti-Rho A and anti-N-cadherin antibodies in 1:200 dilution in 4 °C overnight. The next day, cells were washed three times for 5 min each with 0.05% Triton X-100 solution and then incubated with 1:50 dilution of secondary anti-mouse antibodies conjugated with AlexaFluor® 488 by 2 h in room temperature. After this time, the slides were rinsed with distilled water, the separation chambers have been removed and coverslips were applied. The slides were observed at the *λ* = 488 nm using an Axiovert 200M confocal microscope with an LSM 5 PASCAL scanning head (Zeiss).

### Scanning electron microscopy (SEM)

The cells were exposed to osthole, LY294002 and combination of this compounds for 24 h and fixed with 4% glutaraldehyde and 1% osmium tetroxide at 4 °C for 2 h. Then, the cells were dehydrated in graded acetone (30, 50, 70, 80, 90 and 100%) for 15 min each and critical point dried. To coat the samples with gold, an Emitech K550X Sputter Coater was applied. The samples were examined using a TESCAN vega 3 LMU microscope (Czech Republic).

### Cell migration test

Tumor cell migration was assessed by means of the wound assay model. The cell lines were grown at 2.5 × 10^5^ in standard conditions (37 °C, 95% humidity, 5% CO_2_) in 4-cm-diameter culture dishes (NuncTM, ThermoFisher). Cells were grown to confluence and next scratched with the pipette tip (P300). The medium and dislodged cells were aspirated, and the plates were rinsed twice with PBS. Next, fresh culture medium was added, and after 24 h the number of cells that had migrated to the wound area was estimated, both in control cultures (0.01% DMSO), and incubated with the tested compounds. In addition to the positive control, a negative control (wound) was also prepared and stained immediately after the scratch was made. For this purpose, May-Grünwald-Giemsa staining was used. The observation was performed with the use of a BX51 microscope (Olympus), and the distances between the scratch fronts were estimated using the CellSans program.

### Chou-Talalay method

The concentrations of the compounds used in the combination were determined using the CompuSyn program, using the method developed by Chou and Talalaya (Chou 2010). Based on the number of apoptotic cells (%), the combination index (CI) and dose reduction index (DRI) were calculated. CI < 1, CI = 1 and CI > 1 indicate a synergistic, additive and antagonistic effect, respectively. The DRI represents the fold reduction in the dose of compounds during combination therapy, compared to the concentration of a drug applied alone, producing the same effect. DRI > 1 indicates a reduction in the dose of the drug used in the combination, compared to monotherapy. In addition, in order to determine the optimal dose of treatment, an isobologram was prepared by placing drug doses on both axes of the graph, having the same anticancer activity (IC50, IC75 and IC90). The straight line connecting both points illustrates the additive effect, and the coordinates of the points on it indicate the doses of drugs that exhibit additive effects in combined therapy. Points below the line determine the optimal doses of drugs, assuming synergism of their action, and doses used in an antagonistic interaction should be above the additive effect line.

### Statistical analysis

One-way analysis of variance (ANOVA) test followed by Dunnett’s multiple comparison analysis was used for statistical evaluation. *p* < 0.05 of data presented as mean ± standard deviation (SD) was taken as the criterion of significance.

## Results

### Effect of osthole in combination with LY294002 on the programmed cell death induction

#### Apoptosis, autophagy and necrosis after the treatment with osthole and LY294002

To detect apoptotic, autophagic and necrotic cells, anaplastic astrocytoma (MOGGCCM) and glioblastoma multiforme (T98G) were stained with specific fluorochromes: Hoechst 33342, acridine orange and propidium iodide, respectively.

**Fig. 1:**
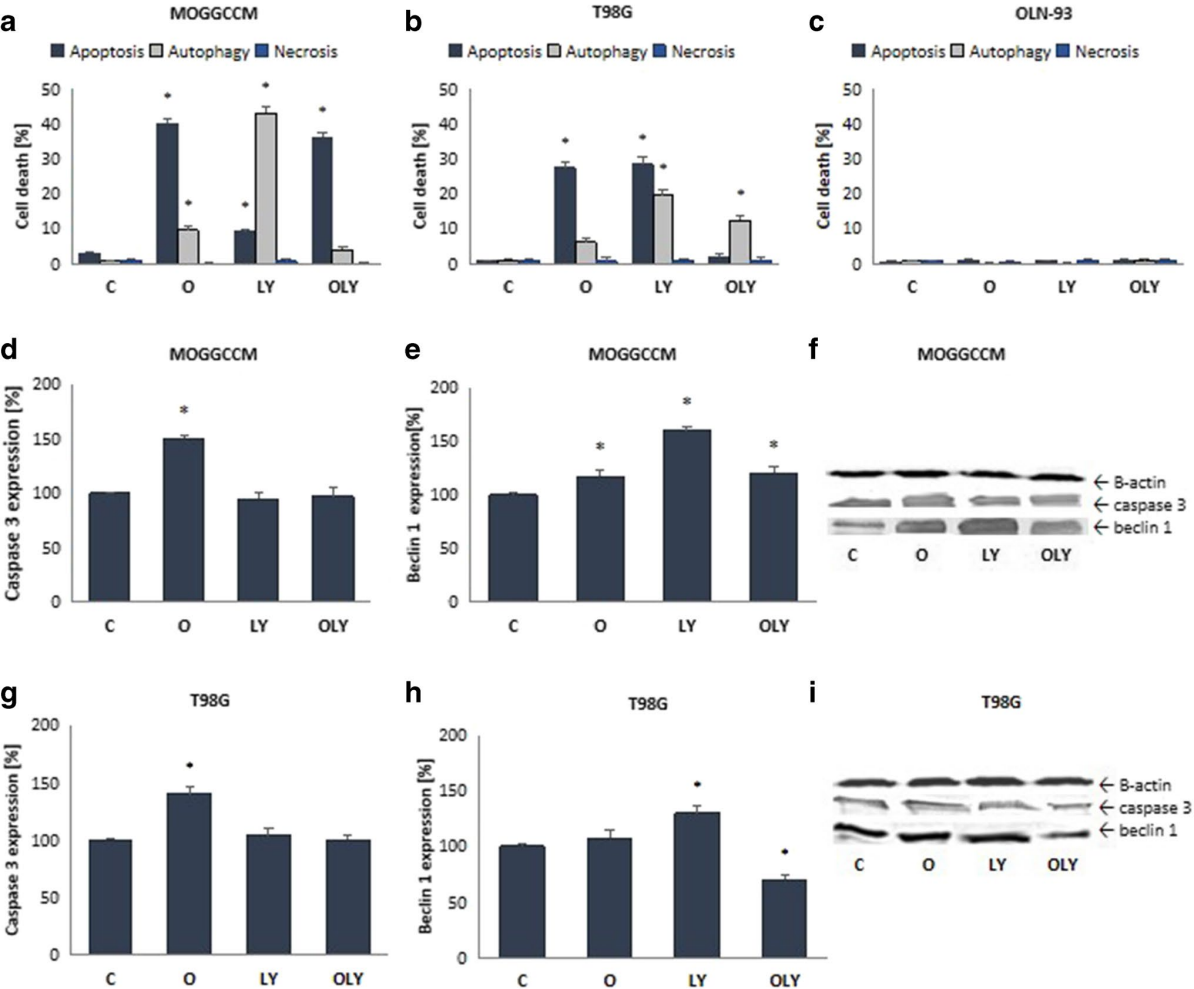
The effect of osthole or/and LY294002 on apoptosis, necrosis and autophagy induction (**A**, **B**, **C**) and the level of caspase 3 (**D**, **G**) and beclin 1 (**E**, **H**) in MOGGCCM (**A**, **D**, **E**, **F**), T98G (**B**, **G**, **H**, **I**) and OLN-93 (**C**) cell lines. C—control, O—osthole, LY—LY294002; *p < 0.05

The results presented at Fig. 1 showed that a single application of osthole was associated with the induction of apoptosis in approx. 40% of AA and 27% of GBM cells. Autophagy was also observed in both cell lines, but it did not exceed 10%. This process turned out to be dominant after incubation of MOGGCCM cells with LY294002, causing the death of 43% MOGGCCM cells. In the case of the T98G line, apoptosis and autophagy accounted for 28% and 20%, respectively. Simultaneous treatment with osthole and LY294002, practically, completely inhibited the ability of the PI3K inhibitor to induce autophagy in MOGGCCM cells. Moreover, combination therapy initiated apoptosis in more than 35%, while LY294002 alone induced the process in non-sleep 10% of cells. Interestingly, glioblastoma cells incubated with a PI3K inhibitor and coumarin showed significant resistance to the induction of both autophagy and apoptosis compared to a single application of LY294002. What is also important, the LY294002 inhibited the pro-apoptotic activity of osthole, reducing the percentage of apoptotic cells by about 15%.

Studies carried out on oligodendrocyte cell line OLN-93 have shown that osthole and LY294002, alone and in combination, do not have cytotoxic properties in relation to normal cells.

### Effect of osthole and LY294002 on the level of cell death marker proteins

Cell death processes are tightly regulated at the molecular level by specific marker proteins. In the case of apoptosis, caspase 3 plays a key role, while autophagy is regulated by beclin 1. In order to confirm the types of cell death observed under the microscope, we measured the amounts of these proteins.

Immunoblotting studies showed that LY294002, alone or in combination with osthole, had no significant effect on cleaved caspase 3 levels, while single application of coumarin significantly increased caspase 3 levels in both cell lines (Fig. 1D, G).

In the case of beclin 1, it was significantly increased in both cell lines after application of the PI3K kinase inhibitor alone (Fig. 1E, H). A similar effect was also obtained after treatment of anaplastic astrocytoma cells with osthole. Interestingly, the combination of both compounds reduced the amount of this protein compared to a single application of LY294002.

### Blocking of beclin 1 and Bcl-2 expression by siRNA

**Fig. 2:**
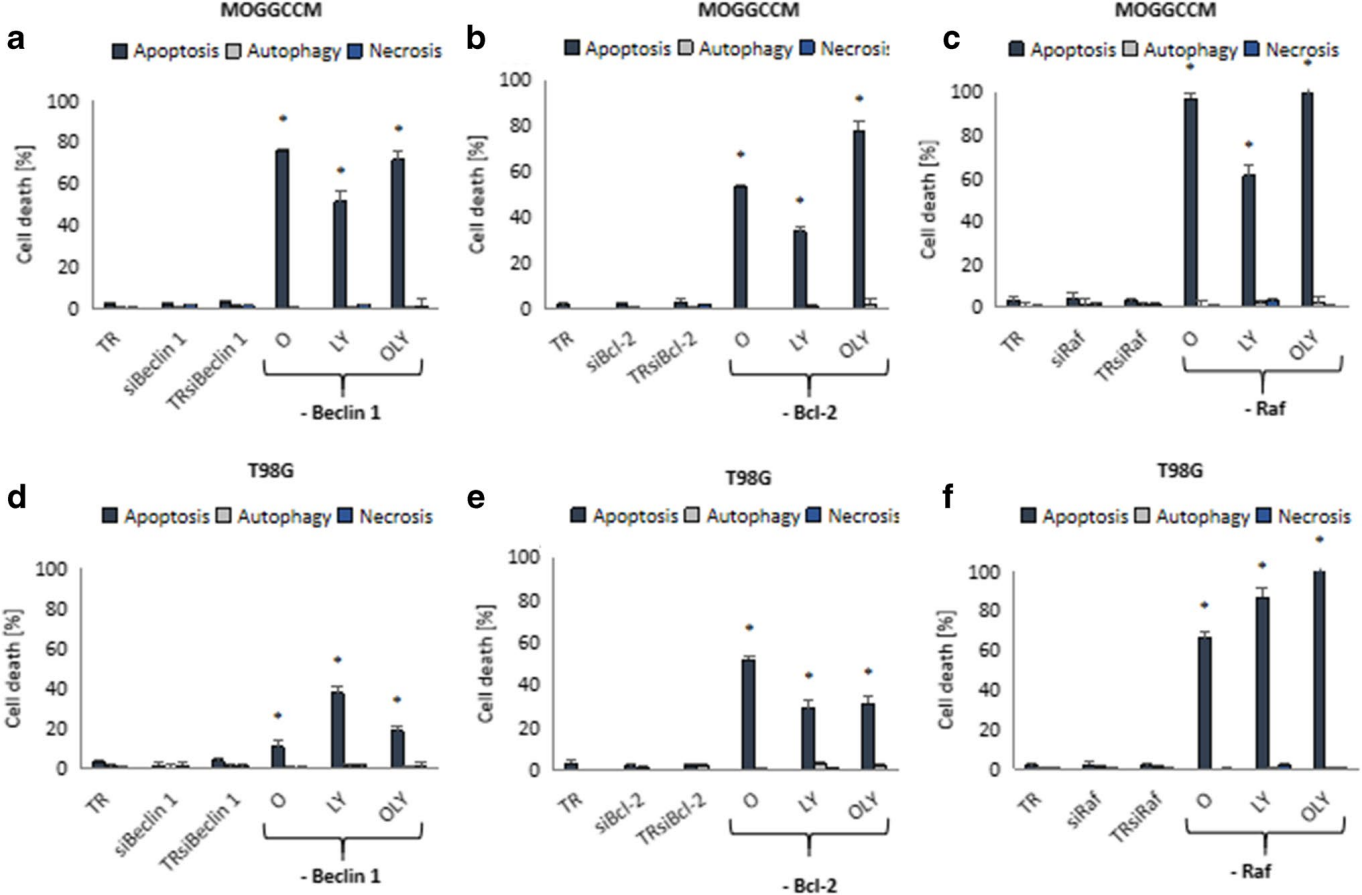
Level of apoptosis, autophagy and necrosis in MOGGCCM (**A**, **B**, **C**) and T98G (**D**, **E**, **F**) cells with silenced beclin 1 (**A**, **D**), Bcl-2 (**B**, **E**) and Raf kinase (**C**, **F**) gene expression by specific siRNA upon osthole (O) and LY294002 (LY) treatment; TR—cells incubated with transfection reagent, siBeclin 1/siBcl-2/siRaf—cells incubated with siBeclin 1, siBcl-2 or siRaf; *p < 0.05

Beclin 1, because of presence of the BH3 domain in its structure, has the ability to interact with proteins that also have this domain. They include, e.g. Bcl-2 protein, which is a kind of molecular switch between the process of apoptosis and autophagy. As it results from the conducted research, blocking the expression of both beclin 1 and Bcl-2 protein completely eliminated the autophagy in anaplastic astrocytoma and glioblastoma multiforme cells (Fig. 2A–D). In the MOGGCCM line, in all experimental variants, increase of apoptosis was observed, with the best results obtained after the simultaneous application of osthole and LY294002 (approx. 80%) (Fig. 2A, B). Transfected T98G cells turned out to be much less sensitive to the induction of apoptosis (Fig. 2D). In this case, the silencing of beclin 1 expression was associated with a decrease in the pro-apoptotic properties of osthole alone, while increasing the effectiveness of combination therapy by approximately 20%. Slightly better results were obtained in cells transferred with siBcl-2 and treated with osthole, alone and in combination with a PI3K inhibitor, achieving apoptosis in 50% and 30% of tumor cells, respectively (Fig. 2E).

### Blocking of Raf kinase expression

According to the literature, malignant transformation of gliomas is associated with excessive activation of the Ras-Raf-MEK-ERK and PI3K-Akt/PKB-mTOR pathways. Therefore, in addition to the PI3K kinase inhibitor, Raf kinase expression was silenced. Microscopic analysis (Fig. 2C, F) showed that cells transfected with siRaf were significantly more sensitive to apoptosis induction by osthole and LY294002. In the MOGCCM line, coumarin (alone and in combination with LY294002) eliminated almost all tumor cells (99%), similarly to T98G after both drug treatment. Osthole and LY294002 alone were less effective, but still the % od apoptotic cells exceeded 60%.

#### Effect of osthole in combination with LY294002 on migration potential

**Fig. 3:**
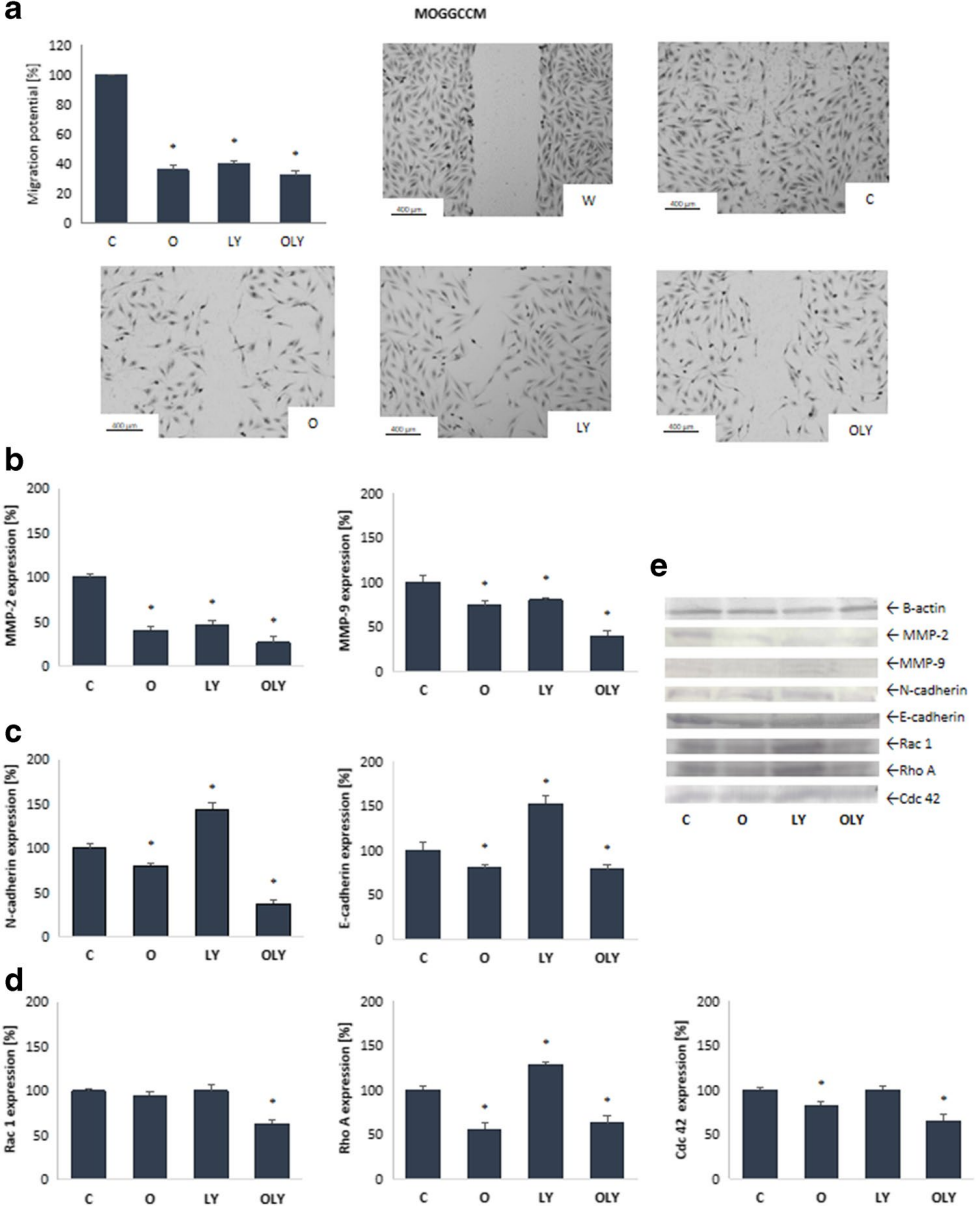
Migration potential and level of metaloproteinases (MMP-2, MMP-9) (**B**, **E**), cadherins (N-cadherin and E-cadherin) (**C**, **E**) and Rho family proteins (Rac 1, Rho A and Cdc 42) (**D**, **E**) in MOGGCCM cells upon osthole or/and LY294002 treatment. W—wound, C—control, O—osthole, LY—LY294002; *p < 0.05

**Fig. 4:**
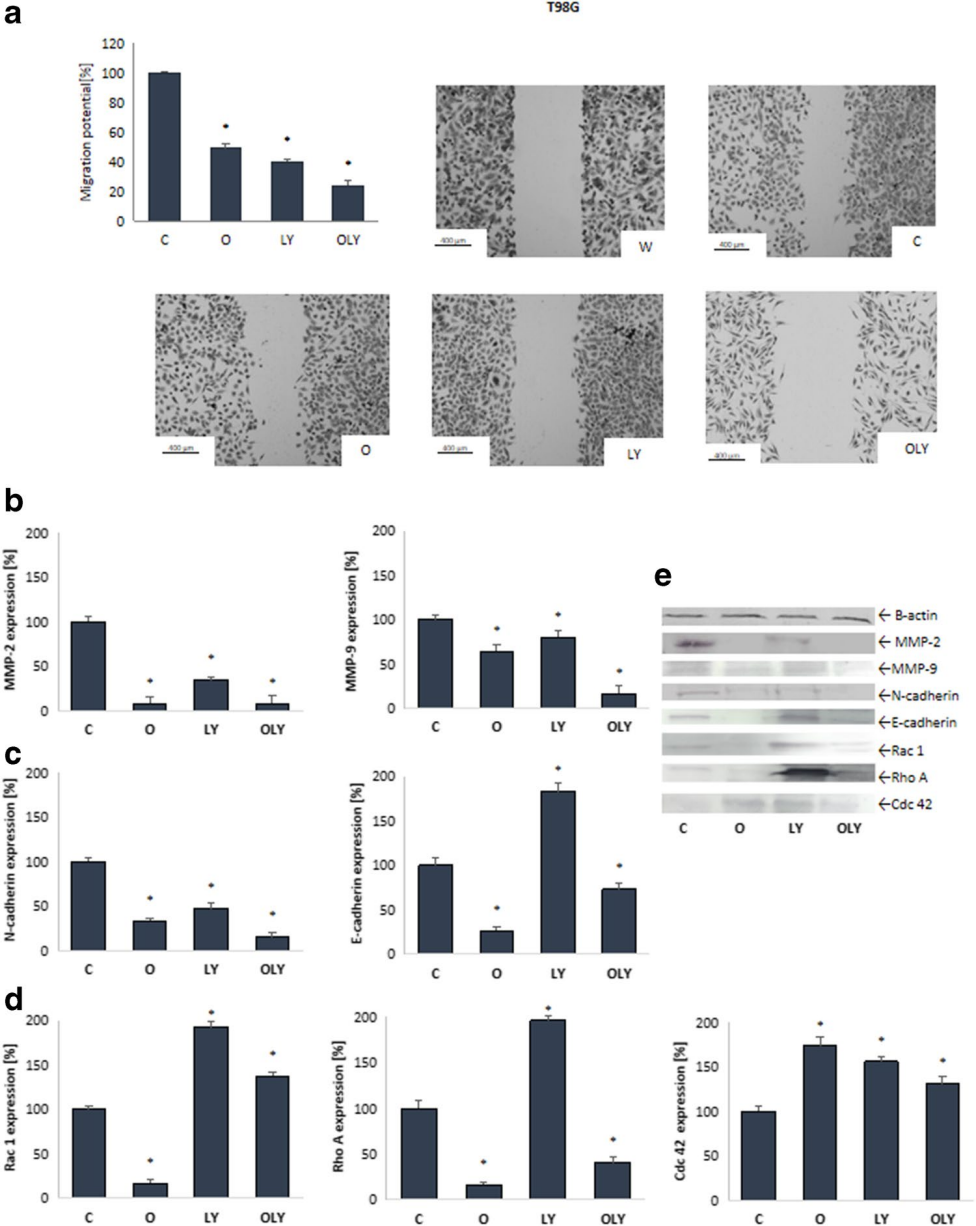
Migration potential and level of metaloproteinases (MMP-2, MMP-9) (**B**, **E**), cadherins (N-cadherin and E-cadherin) (**C**, **E**) and Rho family proteins (Rac 1, Rho A and Cdc 42) (**D**, **E**) in MOGGCCM cells upon osthole or/and LY294002 treatment. W—wound, C—control, O—osthole, LY—LY294002; *p < 0.05

In addition to pro-apoptotic activity, properties that inhibit the migration of cancer cells play a very important role in the development of new pharmacological therapeutic strategies. For this reason, the effect of osthole and LY294002 on the migration potential of glioma cells was investigated using the scratch test (Figs. 3A and 4A). According to the study, both coumarin and LY294002, used alone and in combination, reduced the migration potential of AA and T98G cells by more than 50%. In both cell lines, the most effective was the combined application of coumarin with an inhibitor, which reduced the mobility of anaplastic astrocytoma and glioblastoma multiforme cells by 70 and 75%, respectively.

### Effect of osthole and LY294002 on the level of cadherin, metaloproteinases and Rho family protein

The appearance of a metastasis strictly depends on the migration and invasion potential, which is regulated by changes in the expression of adhesion proteins (E-cadherin and N-cadherin) and metaloproteinases (MMP-2 and MMP-9). A significant role is also played by proteins from the Rho family, which are directly involved in the formation of lamellipodium and filopodium (Rac 1 and Cdc 42) and the polarization of the moving cell (Rho A). Therefore, using the immunoblotting method, the level of these proteins was estimated.

Metalloproteinases (MMP-2 and MMP-9) are involved in the destruction of the extracellular matrix, and their increased expression may increase the migration and invasive potential of cancer cells. The conducted studies showed that osthole and LY294002 reduced the level of the active form of metaloproteinases 2 and 9 (Figs. 3B and 4B). After application of both compounds, alone and in combination, the amount of mature MMP-2 was reduced by at least half. In the case of the MOGGCCM line, the simultaneous application of the compounds turned out to be the most effective, reducing the amount of protein by approx. 75%. Even better results were obtained in the T98G line, where osthole, alone and in combination with the PI3K inhibitor, reduced the level of MMP-2 by more than 90%. In both cell lines, a decrease in the amount of cleaved MMP-9 was also observed at the level of approx. 25% after the application of osthole alone or LY294002. Simultaneous application was significantly more effective and reduced metaloproteinase in AA and GBM cells by 60% and 80%, respectively.

Studies at the molecular level showed that in the MOGGCCM line (Fig. 3C), LY294002 increased the amount of both N-cadherin and E-cadherin by about 50% compared to the control level, while osthole reduced the amount of these proteins. Interestingly, in the case of N-cadherin, the combination of both compounds turned out to be more effective than coumarin alone and reduced the level of the protein by about 60%. The amount of E-cadherin was comparable to osthole alone. Slightly different effects were obtained in glioblastoma multiforme cells (Fig. 4C) In this case, coumarin and an inhibitor (alone and on combination) reduced the level of N-cadherin by more than 50%. Also in this case, the use of simultaneous application turned out to be the most effective. The analysis of the amount of E-cadherin showed its significant increase after the application of LY294092 alone (by about 75%) and a decrease after the simultaneous application of the PI3K inhibitor with osthole (25%). Coumarin alone was even more effective and fourfold reduced protein levels.

As demonstrated by immunoblotting (Fig. 3D), a single application of osthole or LY294002 had no significant effect on Rac 1 protein levels in AA cells, while simultanous aplication reduced its levels by approximately 30%. Interestingly, in the T98G line, only osthole reduced the amount of Rac1, while the use of LY294002, alone and in combination with coumarin, increased the amount of this protein (Fig. 4D). In the case of the Rho A protein, both in anaplastic astrocytoma and glioblastoma multiforme cells, its significant increase was noted after the application of LY294002. Quite the opposite effects were obtained using osthole, which reduced the amount of protein by nearly 50% in AA cells and over 75% in GBM cells compared to the control level. Similar results were obtained using combinations of both compounds. At the same time, it was observed that osthole and LY394002, both in single and combined application, increased the level of Cdc 42 protein in T98G cells, with the largest increase (by approx. 75% compared to the control level) observed after the application of only coumarin. Different results were obtained in AA cells, where incubation with osthole, alone and in combination with LY294002, reduced the amount of Cdc 42, while the inhibitor alone had no effect on the level of this protein.

### The cellular localization of the Cdc 42, Rho A and N-cadherin upon osthole and LY294002 application

**Fig. 5:**
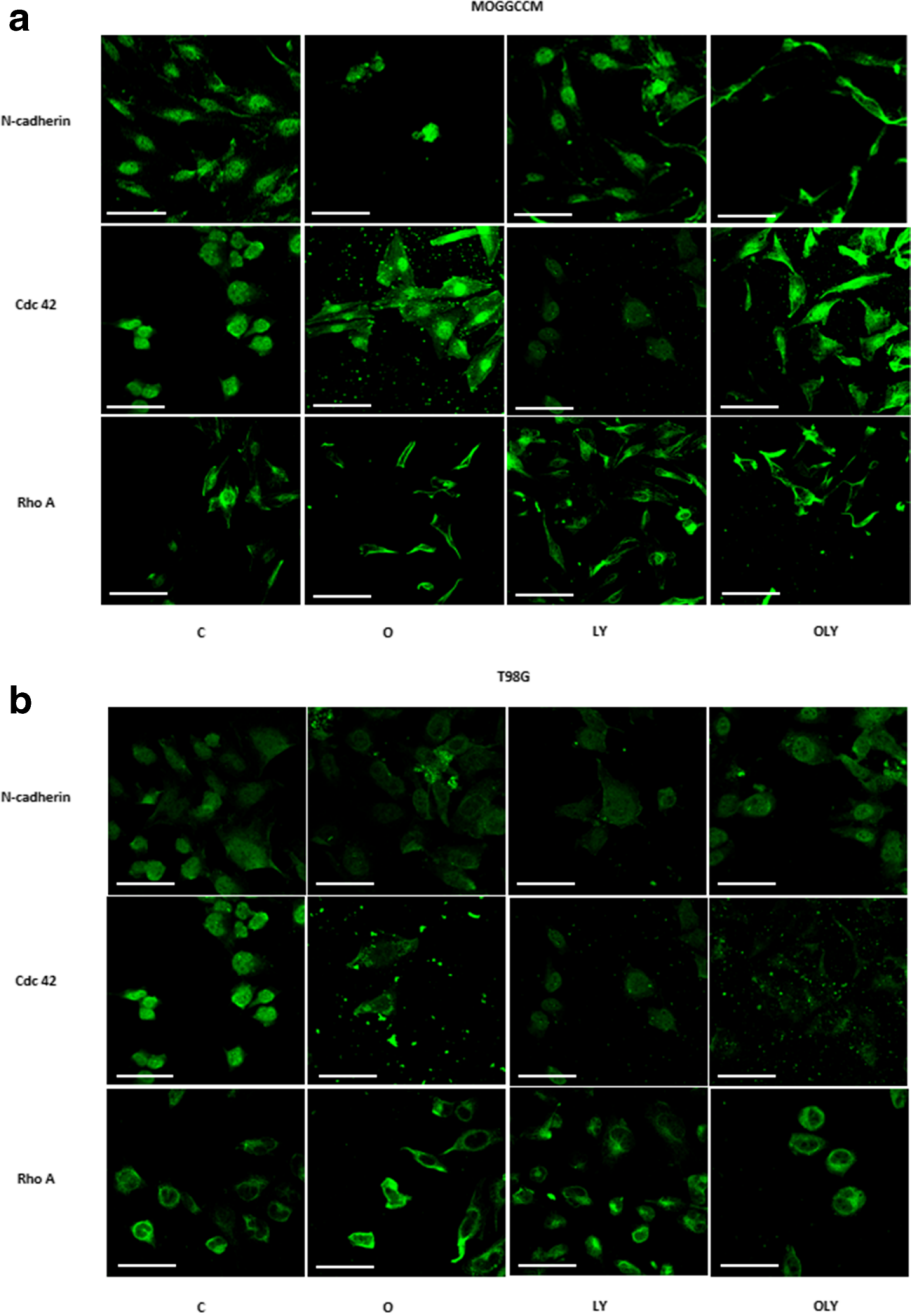
Localization of N-cadherin, Cdc 42 and Rho A in MOGGCCM (**A**) and T98G (**B**) cells after osthole (O) or/and LY294002 (LY) treatment. C—control

In addition to the level and activity, the localization of studied proteins plays a key role. As we have shown (Fig. 5), in both AA and GBM cells, N-cadherin was mainly found within the cell nucleus, while the use of osthole and LY294092, in single and combined application, changed its localization to the cytoplasmic, inclose to cell membrane. In the case of the Cdc 42 protein, in control cells increased fluorescence was noted in the vicinity of the cell nucleus, while coumarin (alone and in application with LY294002) led to the displacement of part of the protein to the cytosol and cell membrane. There was no change in the localization of the Rho A protein, which was located mainly in the cytoplasm.

### Scanning electron microscopy of cells treated with osthole and LY294002

**Fig. 6:**
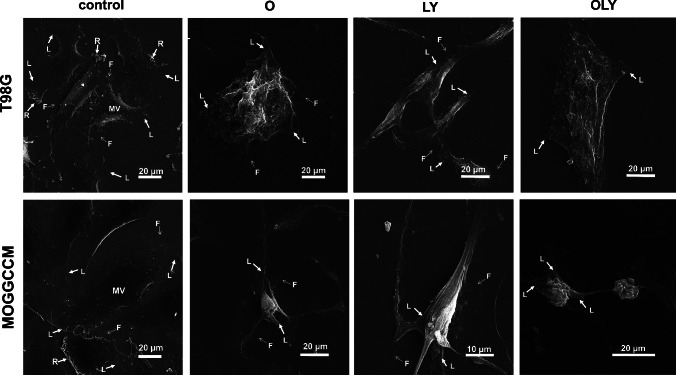
T98G cell morphology examined by scanning electron microscopy (SEM) after osthole (O) and LY294002 (LY) treatment. C—control, L—lamellipodium, F—filopodium, R—membrane ruffles, MV—microvilli

The ability of cells to migrate is determined by the presence of protrusions on their surface: lamellipodia and filopodia. Using the technique of scanning electron microscopy, their presence on the surface of anaplastic astrocytoma and glioblastoma cells was visualized (Fig. 6). Moreover, characteristic dense microvilli and the so-called ruffles membrane were also observed. Interestingly, when AA and GBM cells were incubated with osthole and LY294002 alone and in combination, these structures were absent. The tested compounds additionally shortened and reduced the number of lamellipodia and filopodia, and the combination therapy completely eliminated the filopodia.

### Chou-Talalay method

**Fig. 7:**
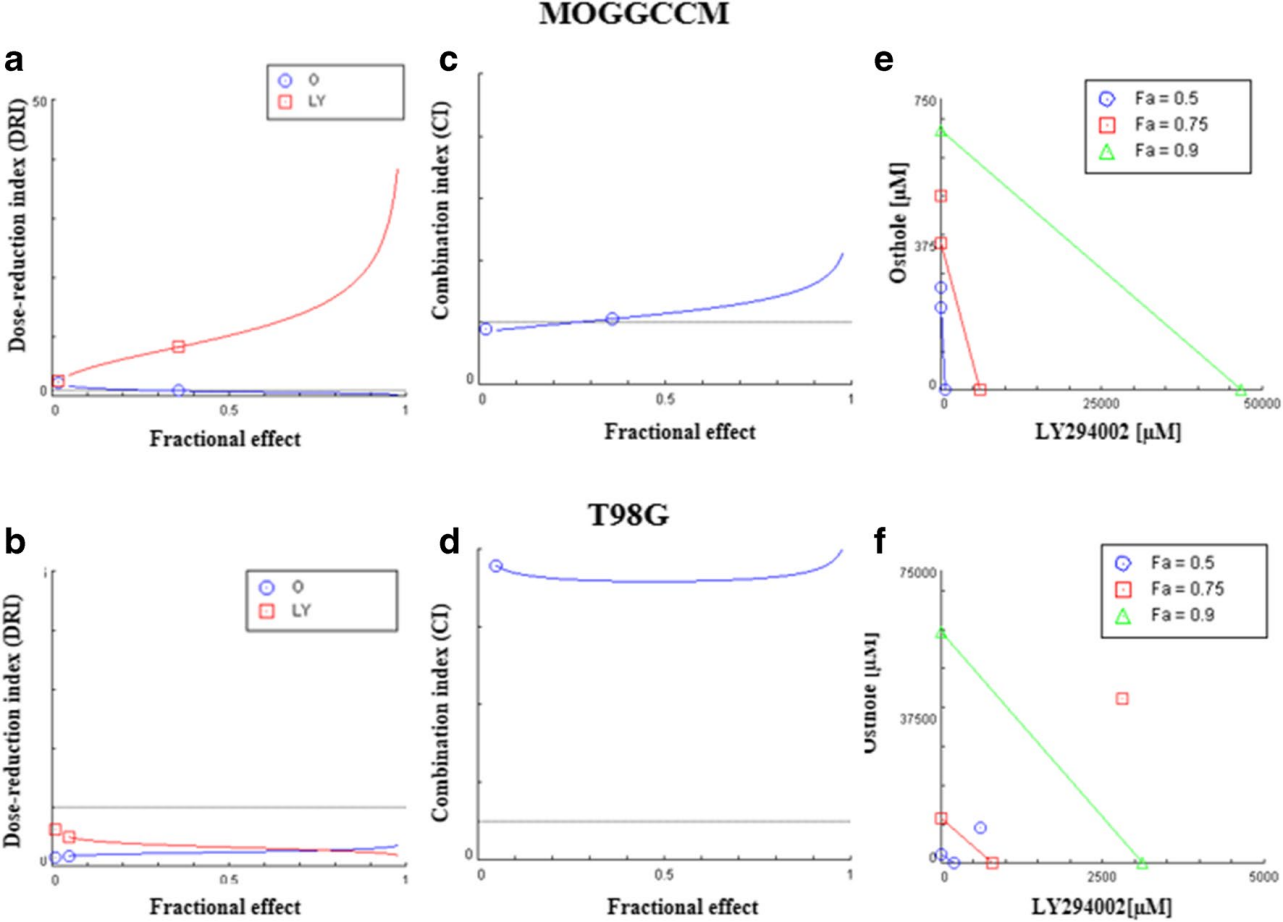
Osthole and LY294002 combination treatment in MOGGCCM (**A**, **C**, **E**) and T98G (**B**, **D**, **F**) cell lines. **A**, **B** Combination index (CI) plot: The combination index is plotted as a function of Fa (fractional effect). **C**, **D** The Fa-DRI (dose reduction index) plot (Chou-Martin plot). **E**, **F** Isobologram for combination: Classic isobologram at IC50, IC75 and IC90

In addition to the experimental method, the effectiveness of the combined treatment was assessed using the Chou-Talalay method. In the performed test, the ability of compounds to induce apoptosis was taken into account. The obtained results indicated the antagonism between osthole and LY294002, both in the MOGGCCM and T98G cell lines (Fig. 7A, B). However, in AA cells, with lower drug effects (IC > 20%), this combination was synergistic or additive. The obtained results correlated with the DRI value (DRI < 1), and the concentration of osthole and LY294002, giving the same pro-apoptotic effect, should be higher with simultaneous application compounds (Fig. 7C, D). In addition, isobolographic analysis showed that the concentrations of drugs used in a single application gave an optimal effect also in combination therapy (Fig. 7E, F).

## Discussion

Gliomas are tumors of the central nervous system with an extremely poor prognosis. The currently used methods of treatment allow only to extend the patient’s life and improve its quality. Due to the infiltrative nature of the tumors, their complete surgical resection is practically impossible, and in the course of radio- and chemotherapy, treatment resistance is very often acquired. Therefore, it is extremely important to develop new therapeutic strategies that can support the currently used forms of treatment. Huge application potential in this aspect has osthole. The anticancer activity of this coumarin has been demonstrated in in vitro studies using lung cancer cells (A549), gastric cancer cells (HGC27 and SGC-7901), leukemia cells (P-388 D1), breast cancer cells (MCF-7, MDA-MB 231, MDA-BT-549, MDA-MB-468 and MDA -MB 435), medulloblastoma (TE671), laryngeal cancer (RK33), cervical cancer (HeLa) and glioblastoma (MOGGCCM, T98G) (Dai et al. 2018; Xu et al. 2018, 2011; Wang et al. 2015; Jarząb et al. 2014; Yang et al. 2010; Chou et al. 2007).

As it is known from the literature data, in the vast majority of gliomas, changes in signal transmission from the cell membrane to the nucleus through intracellular pathways, such as PI3K-AKT/PKB-mTOR, are observed (Ludwig and Kornblum 2017). Therefore, the use of molecularly targeted therapy seems to be a promising form of treatment. It has been described that blocking PI3K-AKT/PKB-mTOR transmission with specific inhibitors favorably increases the sensitivity of glioma cells to the induction of programmed death. This applies to, in particular, the combination therapy, especially with natural compounds, which enhances the anticancer potential of clinically used treatment (Jakubowicz-Gil 2009; Schwartzbaum et al. 2006). Therefore, apart from a single application of osthole, its combination with LY294002—an inhibitor of PI3K kinase—was used.

Osthole, in a separate application and in combination with LY294002, eliminated almost 40% of AA cells by apoptosis, while the PI3K inhibitor alone dominantly induced autophagy (approx. 45%). In the case of T98G cell line, the dominant type of death induced by coumarin alone was apoptosis (approximately 30%), while LY294002 caused both apoptosis and autophagy at 30% and 20%, respectively. Interestingly, combination therapy in GBM cells turned out to be antagonistic, completely inhibiting the pro-apoptotic potential of osthole and reducing the pro-autophagal properties of LY294002 to about 10%. In the light of recent reports on the possibility of using autophagy as a survival mechanism by cancer cells, reducing the level of the process seems to be highly desirable (Jin and White 2008). It has been observed that the inhibition of the autophagy significantly increases the effectiveness of anticancer therapies (Amaravadi et al. 2019). This process may also be responsible for developing resistance to the chemotherapeutic agents used, e.g. by protecting cancer cells against programmed death (Lim and Murthy 2020), which would explain the much weaker pro-apoptotic effect of the combination of osthole with LY294002, compared to a single application of coumarin. Studies at the molecular level have shown that the pro-autophagal activity of the PI3K inhibitor was associated with an increased level of beclin 1, and the induction of apoptosis by osthole was accompanied by an increased level of cleaved caspase 3. This enzyme, in addition to regulating of apoptosis, has the ability to proteolyze beclin 1 into two fragments: N-terminal and C-terminal. N-terminal fragments, devoid of the so-called nuclear export signal, are located in the nucleus and have a BH3 domain, capable of binding anti-apoptotic proteins. In turn, the C-terminal fragments move to the mitochondria, leading to the release of cytochrome c and induction of apoptosis (Wirawan et al. 2010; Zhu et al. 2010). In AA cells incubated simultaneously with osthole and LY294002, despite the increased level of beclin 1, autophagy was not observed, which was present when each compound was applied separately. In this case, the presence of functional caspase 3 could change the properties of beclin 1 from pro-autophagal to pro-apoptotic. This may explain the significantly higher percentage of apoptotic cells, despite the increased level of the autophagy marker. The key role of beclin 1 in the induction of autophagy was confirmed by the inhibition of protein expression with a specific siRNA. Autophagy was not observed in the transfected AA and GBM cells. Interestingly, anaplastic astrocytoma showed a significant increase in the number of apoptotic cells (> 50%) after treatment with either coumarin or LY294002, alone and in combination. In the T98G line, such an increase was noted after application of LY294002 (alone and in combination with osthole), while incubation of the transfected cells with only coumarin significantly reduced the pro-apoptotic activity. It has been shown that beclin 1 has the ability to bind to anti-apoptotic proteins, including the Bcl-2. The presence of the protein complex prevents the induction of autophagy, while promoting apoptosis (Marquez and Xu 2012). As our previous research has shown, coumarin leads to the formation of this complex (Sumorek-Wiadro et al. 2020a). Thus, inhibition of beclin 1 expression is associated with an increase in the amount of anti-apoptotic protein Bcl-2, which inhibits apoptosis, and this explains the results obtained. Moreover, blocking the expression of the Bcl-2 protein significantly increased the effectiveness of the compounds used, both in single and combined application. These results are consistent with other reports, according to which increased expression of the gene encoding Bcl-2 in gliomas cells leads to an increase in drug resistance and a decrease in the apoptotic response in vitro (Garcia-Aranda et al. 2018). Our previous studies have shown that the use of an inhibitor of the Ras-Raf-MEK-ERK pathway increased the effectiveness of the osthole treatment (Sumorek-Wiadro et al. 2020b). As we have shown, even better effects are obtained by blocking Raf kinase expression. Well, simultaneous incubation of transfected AA and GBM cells with coumarin and LY294002 induced apoptosis in all cells. The observed changes confirm the huge role of the excessive activity of the RAS-RAF-MEK-ERK and PI3K-Akt/PKB-mTOR pathways in the resistance of gliomas to the applied therapy (Steelman et al. 2011).

Excess mortality and difficulties in the treatment of gliomas are associated with their potential for rapid, infiltrative growth and the formation of distant metastases (Armento eta al., 2017; Langhans et al. 2017). Therefore, compounds that inhibit the migration of cancer cells are of great importance when designing new therapeutic strategies. From the literature data it is known that the predictor of tumor cell mobility is the increased activity of PI3K and AKT/PKB kinases. It has been shown that these proteins stimulate the secretion of metalloproteinases, responsible for the degradation of the extracellular matrix. In addition, by reducing the level of E-cadherins, they facilitate the epithelial-mesenchymal transition (Chin and Toker 2009; Qiao et al. 2008; Kim et al. 2001). Therefore, PI3K inhibitors have a great application value. As shown by Chen et al., LY294002, by reducing the level of PI3K and AKT/PKB activity, significantly reduced the invasion of U87 cells (GBM) (Chen et al. 2012). Similar effects were obtained in the conducted studies, where both osthole and LY294002, alone and in combination, inhibited the migration potential of anaplastic astrocytoma and glioblastoma multiforme cells, with the best effects obtained after the simultaneous application of both compounds.

The invasive potential of cells depends to a large extent on their ability to degrade the extracellular matrix by the secretion of metaloproteinasess (MMPs). Removal of the spatial barrier by MMP allows cancer cells to move and form metastases (Visse and Nagase 2003). So far, in gliomas, increased expression of MMP-2 and MMP-9 has been described, correlating with the degree of tumor malignancy (Rao 2003; Choe et al. 2002). As we have shown in our studies, ostol and LY294002, in single and combined application, led to a decrease in the level of active form of these proteins. Coumarin reduced the expression of genes encoding the mentioned MMPs also in breast, ovarian and lung cancer (Jiang et al. 2016; Xu et al. 2012; Yang et al. 2010). An analogous way of inhibiting migration was noted in retinal endothelial cells treated with LY294002 (Di and Chen 2018).

Another group of proteins directly involved in the migration of cancer cells are cadherins. These proteins belong to the superfamily of calcium-dependent adhesion proteins that enable the epithelial-mesenchymal transition. In the vast majority of cancers, reduced expression of E-cadherin and increased expression of N-cadherin are observed; however, in the case of gliomas, the prognostic significance of the expression of these proteins remains unexplained. Gliomas by nature do not have epithelial phenotypes, and changes in E-cadherin expression are extremely rare. The key role seems to be played by neuronal cadherin (N-cadherin), which is overexpressed in the vast majority of tumors. Interestingly, recent studies have shown that the mesenchymal phenotype of glioma cells determines their increased therapeutic resistance. However, data on the effect of N-cadherin on invasive potential are conflicting. Some of them indicate that the reduction of N-cadherin expression increases the mobility of cancer cells, while others suggest that an increased level of the protein may correlate with reduced survival (Kim et al. 2023; Osuka et al. 2021; Noronha et al. 2021; Noah et al. 2017). Our results indicate that osthole, alone and in combination with LY294002, significantly reduced the level of N-cadherin in both cell lines, and the protein was located mainly in the cell membrane, being responsible for cell-cell adhesion. Similar effects were obtained after the application of the PI3K inhibitor in the T98G line, while incubation of AA cells increased level of the protein, which was mainly found in the nucleus. Nuclear and cytoplasmic localization of N-cadherin in control AA and GBM cells may indicate the involvement of N-cadherin in the regulation of intracellular signaling pathways (Radice 2013). Osthole (alone and in combination with LY294002) also decreased E-cadherin levels, while the PI3K inhibitor alone increased the protein levels. However, due to the highly questionable protective role of E-cadherin in gliomas, the effects obtained after a single application of LY294002 are not entirely desirable. It has been shown that the increase in E-cadherin expression in higher-grade gliomas correlates with increased invasive potential and worse prognosis for patients (Noronha et al. 2021).

The movement of the cell depends on its polarization, which is possible due to the gradient distribution of signaling proteins, which include small GTPases from the Rho family, such as Cdc 42, Rac 1 and Rho A (Osmani et al. 2010). High expression of Cdc 42 in GBM is correlated with lower survival rates (Al-Koussa et al. 2020; Okura et al. 2016). We have shown that osthole and LY294002 decreased the level of this protein in anaplastic asteroma cells, which correlated with a reduced number of shorter filopodia. The best results were obtained after using combination therapy. At that time, the amount of protein was the lowest, and no filopodia were visible in the scanning microscopy image. There were no significant changes in the localization of the protein compared to the control group. Completely different results were obtained in the case of glioblastoma multiforme, where both single and combined application of the compounds increased Cdc 42 levels. The localization of the protein was also changed, which in this case was observed in numerous vesicles present in the cytosol. In the microscopic image, shorter (after application of coumarin or LY294002) or complete lack of filipodia (after simultaneous application of compounds) was noticed. One possible explanation for the lack of correlation in this aspect is the presence of the protein in an inactive form.

Another protein responsible for the formation of protrusions is Rac 1. As we showed, its level in T98G cells decreased after application of osthole alone, while in the MOGGCCM cell line, combined application was the most effective. The obtained results were confirmed by scanning microscopy, where a reduced number of shorter lamellipodia was observed. In addition, osthole, in a single application and in combination with LY294002, eliminated the occurrence of the membrane ruffles, characteristic of invasive cells, which correlated with a decrease in the amount of Rho A protein (Kurokawa and Matsuda 2005). Interestingly, the PI3K inhibitor (alone and with osthole) significantly increased the amount of Rac 1 in GBM cells, but did not affect the formation of spikes. In addition, the application of the compound also increased the amount of Rho A protein, which also did not correlate with the presence of ruffles membrane. It has been shown that the expression of constitutively active Rho A alone is not sufficient to cause membrane ruffles in HeLa and MDCK cells, and their presence is associated with the presence of active Cdc 42 and Rac 1 (Kurokawa and Matsuda 2005).

## Conclusions

In summary, the described, new combination of compounds shows a high pro-apoptotic potential, depending on the stage of the cancer, and also inhibits the migration of gliomas cells. Taking into account the unusual invasiveness and the degree of metastasis of these tumors, the obtained results may be the basis for the development of new therapeutic strategies, supporting the currently used methods.

## Data Availability

No datasets were generated or analysed during the current study.
